# Perspectives on reasons for suicidal behaviour and recommendations for suicide prevention in Kenya: qualitative study

**DOI:** 10.1192/bjo.2023.7

**Published:** 2023-02-17

**Authors:** Linnet Ongeri, Miriam Nyawira, Symon M. Kariuki, Mary Bitta, Chris Schubart, Brenda W. J. H. Penninx, Charles R. J. C. Newton, Joeri K. Tijdink

**Affiliations:** Centre for Clinical Research, Kenya Medical Research Institute, Nairobi, Kenya; Neuroscience Unit, Kenya Medical Research Institute–Wellcome Trust Research Programme, Nairobi, Kenya; Neuroscience Unit, Kenya Medical Research Institute–Wellcome Trust Research Programme, Nairobi, Kenya; and Department of Public Health, Pwani University, Nairobi, Kenya; Neuroscience Unit, Kenya Medical Research Institute–Wellcome Trust Research Programme, Nairobi, Kenya; and Department of Psychiatry, University of Oxford, UK; Division of Mental Health, Tergooi Medical Centre, Hilversum, The Netherlands; Department of Psychiatry, Amsterdam UMC, Vrije Universiteit, The Netherlands; Department of Ethics, Law and Humanities, Amsterdam UMC, Vrije Universiteit, The Netherlands; and Department of Philosophy, Faculty of Humanities, VU Universiteit, The Netherlands

**Keywords:** Community mental health teams, self-harm, psychiatry and law, suicide, qualitative research

## Abstract

**Background:**

Little is known about the reasons for suicidal behaviour in Africa, and communities’ perception of suicide prevention. A contextualised understanding of these reasons is important in guiding the implementation of potential suicide prevention interventions in specific settings.

**Aims:**

To understand ideas, experiences and opinions on reasons contributing to suicidal behaviour in the Coast region of Kenya, and provide recommendations for suicide prevention.

**Method:**

We conducted a qualitative study with various groups of key informants residing in the Coast region of Kenya, using in-depth interviews. Audio-recorded interviews were transcribed and translated from the local language before thematic inductive content analysis.

**Results:**

From the 25 in-depth interviews, we identified four key themes as reasons given for suicidal behaviour: interpersonal and relationship problems, financial and economic difficulties, mental health conditions and religious and cultural influences. These reasons were observed to be interrelated with each other and well-aligned to the suggested recommendations for suicide prevention. We found six key recommendations from our thematic content analysis: (a) increasing access to counselling and social support, (b) improving mental health awareness and skills training, (c) restriction of suicide means, (d) decriminalisation of suicide, (e) economic and education empowerment and (f) encouraging religion and spirituality.

**Conclusions:**

The reasons for suicidal behaviour are comparable with high-income countries, but suggested prevention strategies are more contextualised to our setting. A multifaceted approach in preventing suicide in (coastal) Kenya is warranted based on the varied reasons suggested. Community-based interventions will likely improve and increase access to suicide prevention in this study area.

## Suicide epidemiology

Suicide is a major public health concern globally. The global age-standardised suicide rate for 2019 was 9.0 per 100 000 population. According to the World Health Organization (WHO) age-standardised 2019 suicide estimates report, the African region carried the greatest suicide burden at 11.2 per 100 000 population, followed by Europe at 10.5 per 100 000 population.^1^ This high suicide rate in Africa has been partly attributed to inadequate investment in the provision of mental health services in the continent.^2^ Kenya's suicide rates mirror the high estimates for Africa, at 11.0 per 100 000 population.^1^ As a way of addressing this high burden of suicide, Kenya is increasingly prioritising suicide prevention mainly at policy level, through public policies that target decriminalisation of suicide in Kenya and the development of a national suicide prevention strategy.^3^ To successfully implement the goals and objectives of these policies, a contextualised knowledge on drivers of suicide in a specific population is necessary.^4^ Understanding communities’ perspective on the risk factors for suicidal behaviour in a specific population is imperative in prioritising and designing targeted interventions and policies for suicide prevention. In addition, a community's suggestions on recommendations for suicide prevention, in line with existing reasons, can inform culturally appropriate and acceptable interventions. However, despite the high burden of suicide in Kenya, no published local study has examined perspectives, experiences and opinions related to the reasons for suicidal behaviour, as well as preference for suicide prevention interventions, from the perspective of various key stakeholders.

## Risk factors for suicidal behaviour

Few studies in Kenya have examined risk factors for suicidal behaviour. Many of these studies have been quantitative surveys, thereby lacking explanations and an in-depth understanding behind these factors.^4,5^ Some suicide risk factors repeatedly highlighted in these quantitative studies include having a mental health condition (primarily depression and substance use disorders),^5^ sociodemographic factors (e.g. male gender for completed suicides^6^ and female gender for non-fatal suicidal behaviour)^7^ and younger age.^6^ Elsewhere, both genetic factors^8^ and personality factors such as neuroticism^9^ have been linked to a high suicide risk. Factors related to a low socioeconomic status have additionally been reported.^6^ One qualitative study conducted in the eastern region of Kenya reported poverty, intimate partner violence, family rejection, social isolation and stigma, as well as chronic physical illness, as factors contributing to suicidal behaviour,^10^ which suggests that multiple factors are involved and may even modify or interact with each other. This informative study, however, only explored perceptions in a pregnant adolescent cohort, and so some themes may not be generalisable to a more diverse population cohort. Further, the study failed to explore opinions on recommendations for suicide prevention interventions.

## Suicide prevention

Recently, the WHO released an implementation guide for suicide prevention, dubbed ‘LIVE LIFE’, in a bid to assist countries in meeting the Sustainable Development Goal of reducing global suicide mortality rate by a third by 2030.^4^ In this guide, four recommendations were put forth: limiting access to means to die by suicide, responsible media reporting, fostering life skills in adolescents and increasing access to care for affected individuals that are expressing suicidal behaviour. These are important evidence-based recommendations and likely to have an impact if implemented by countries. However, to prioritise and inform the implementation process, a contextualised approach of the specific setting of implementation is key.^4^ Gaining a deeper understanding on people's perspectives on what recommendations are best to implement in the setting, in which way should they be implemented and who best to target can provide implementers with useful information on acceptable and culturally appropriate interventions.^11^

Earlier work with this same cohort specifically explored social cultural conceptualisation of suicidality in this population.^12^ In this paper, we report on overall reasons influencing suicidal behaviour in the Coast regionof Kenya, with a focus on recommendations for suicide prevention based on opinions, experiences and perspectives of persons residing in this region. Our study was situated in the Coast region of Kenya, where both incidence and prevalence of suicidal behaviour is known to be high;^6,13^ the treatment gap for mental disorders is large and compounded by the absence of a universal healthcare coverage;^14^ qualitative, in-depth understanding of suicide is lacking^15^ and suicide is unfortunately still criminalised.^16^

## Method

### Ethics statement

The authors assert that all procedures contributing to this work comply with the ethical standards of the relevant national and institutional committees on human experimentation and with the Helsinki Declaration of 1975, as revised in 2008. All procedures involving human patients were approved by the Kenya Medical Research Institute (KEMRI) Scientific Ethics Review Unit (approval number 3916). To maintain confidentiality, all interviews were anonymised. Pseudonyms were assigned and any attribution was made to the category from which the respondents were recruited.

### Study site and participant selection

The study was conducted in Kilifi and Mombasa counties, located on the Indian Ocean coast of Kenya. Kilifi County has seven subcounties, with a population of 704 089 males and 749 673 females, as per the 2019 Kenya Population and Housing Census.^17^ Potential participants from Kilifi County were recruited from within the Kilifi Health and Demographic Surveillance System, coordinated with the KEMRI–Wellcome Trust Research Programme in Kilifi. The surveillance system captures most patients admitted to Kilifi County Hospital, the main referral hospital in the county.^18^ Most residents in Kilifi County are Giriama, a subgroup of the Mijikenda ethnic group. Kilifi County has high illiteracy levels and a high school drop-out rate (34%) reported mostly among girls, because of teenage pregnancies and early marriages.^19^ It is also one of the counties with the highest poverty levels countrywide.^20^ Residents mainly practice Christianity and Islam at approximately equal proportions, with traditional religions also being followed, especially in inland rural areas.^21^

Mombasa, located 65 km away from Kilifi, is the country's oldest and second-largest city, with an estimated population of about 1.2 million people in 2019.^22^ The main ethnic communities found in Mombasa County are the Mijikenda, Swahili and Kenyan Arabs, with Mijikenda being the largest community. As relates to mental healthcare, the Port Reitz mental health facility located in Mombasa County is the only admitting psychiatry hospital in the Coast region.

### Study design and participant selection

Data collection for this qualitative study was conducted through in-depth interviews with key informants between 31 January 2020 and 24 November 2020.

Participants had to be residents of the Coast region of Kenya, willing to provide informed consent and able to communicate in either the local Swahili language or English, to meet the inclusion criteria. We purposively sampled participants with lived experience as suicide survivors or bereaved family members, as well as persons who directly provided care support, law enforcement and judicial services to suicidal persons.

We thus approached front-line healthcare workers with experience of managing cases of suicidal behaviour, persons known to have attempted suicide, local administrative leaders and the judiciary (police officer, chief and magistrate), clergy leaders and bereaved family members of persons who had died of suicide. This wide purposive selection of potential participants was guided by the need to understand a broader perspective, opinions and experiences based on their first-hand knowledge and understanding, being either a person with a history of suicidal attempt or a care and service provider for suicidal victims. Some study participants (e.g. bereaved family members and persons with history of suicide attempt) were identified through collaboration and guidance from the local community leaders, such as the area chief, and from healthcare workers in hospitals in Kilifi and Mombasa Counties. We thus received names and contact details via these healthcare workers after the potential study participants had agreed to be contacted by our team. The traditional health practitioners and the clergy leaders were identified through an existing research database,^21^ whereby they had indicated their willingness to be contacted for future studies.

We approached potential participants in person, provided an overview of the study and invited them to go through the informed consent process to obtain a more detailed understanding of the study goal and activities. Patient participants and bereaved family members were linked through their healthcare providers, and interviews were conducted within the health facilities. Healthcare workers, local administrative leaders, traditional health practitioners and clergy leaders were approached at their workplace, and interviews were conducted in a private space at the same venue. Only the study participant and researchers were present during the interviews. The study information, including participant information and audio recordings, was kept confidential and only accessible by study staff.

A total of 44 potential study participants were purposively selected to participate in the study. Out of these, 19 refused to participate. Reasons for refusal varied, with the majority (*n* = 13) citing time constraints. Table 1 provides the number and gender of study participants in the various categories.

**Table 1:** Composition of participants interviewed

Participants	Number of males	Number of females	Total
Persons with history of suicide attempt	2	2	4
Bereaved family members	5	3	8^a^
Healthcare providers (doctors, nurses, clinical officers, counsellors, social worker)	5	4	9
Religious leaders (Imam, Pentecostal priest, catholic priest)	3	0	3
Traditional healers	2	0	2
Local administration officers (chief, magistrate, police officer)	2	1	3

a. One person with a history of suicide attempt, one religious leader and two healthcare providers are also bereaved family members.

### Study procedures

The interviews were conducted by authors L.O. (research psychiatrist, MMED Psych) and M.N. (research nurse, BScN, PGDip), both female researchers with experience in mental health and training in qualitative methods.

Participants were approached in person. The interviewers provided information about the study objectives, inclusion criteria and why they were interested in the research topic. Participants were then invited to go through the consenting process. An informed consent form can be found in Supplementary Appendix 1 available at https://doi.org/10.1192/bjo.2023.7, which entailed a detailed description of the study. Only participants who gave written informed consent to participate were interviewed. Bereaved family members were interviewed a minimum of 3 months following the suicide bereavement, to allow some time for the grieving process. The shortest interview lasted 16 min, (male, age 20s, bereaved family member), and the longest interview lasted 1 h 27 min (male, age 50s, social worker and bereaved family member).

During the interviews, only the individual participants and researchers were present. Participants had the option of having someone else of their choice present, but none of them requested this. In-depth interviews with persons with history of suicide attempt and bereaved family members were conducted at designated private spaces within health facilities in the two counties. All interviews were conducted face to face in English or Swahili and, in some cases, both languages, depending on the interviewee's preference. All responses were audio-recorded and these recordings were kept confidential and secured.

The interviews entailed administering open-ended guide questions. These were included in a pilot-tested, semi-structured interview guide developed for the study (see Supplementary Appendix 2). First, sociodemographic characteristics were obtained. Subsequently, context-specific exploration of the participants’ experiences and perceptions on suicidality was done. Experiences and perceptions on risk factors for suicidality and suicide prevention were then explored, probing for emerging themes. This paper reports on the risk factors and suicide prevention recommendation that were part of the interview guide. An earlier published paper reported on cultural context-specific exploration.^12^

At the end of each interview, the participants were invited to give any additional comments or ask questions that were not covered in the interview. These comments were captured as part of the audio recordings. Findings from each interview were discussed with the individual participant at the end of each interview, and any clarifications sought. Post-interview comments were recorded by the interviewers in field notebooks immediately after the interview. These comprised details of their feelings, interpretations and other comments. Following discussions by the study team during the data collection phase, it was agreed that after 25 interviews no new ideas, views, perceptions or experiences were being expressed. Data collection was thus stopped at this point of saturation.

A non-judgemental approach was maintained in all of the interviews. The interviewers were keen to listen to and accept the reality of participants’ pain by not trivialising their pain and distress. In some cases, the interviewers provided immediate psychosocial help to participants found to be in extreme distress during data collection. Referral and linkages to care for continued support were then provided. The study had established referral pathways in collaboration with psychologists and clinicians in facilities in the study site.

### Data management and analysis

All audio recordings were transcribed verbatim, and interviews conducted in Kiswahili were translated into English by a bilingual research nurse (Cyrus Theuri (C.T.)) before transcription. Transcripts were not returned to participants because of fieldwork restrictions related to the COVID-19 pandemic, but three members of the study team (L.O., M.N. and C.T.) listened to the audio recordings and examined the transcripts thoroughly for any discrepancies. No repeat interviews were warranted. Qualitative analysis was conducted with both inductive and deductive theme identification.

NVivo software version 10 for Windows (2014, QSR International Pty Ltd, Denver, CO, USA; https://lumivero.com/products/nvivo/) was used for data management. Following familiarisation of the transcribed data, a coding schema was developed, informed by the key research questions, and was iteratively revised by adding new codes that reflected additional themes and topics that were generated from the data. The codes were then systematically applied across all the transcripts, using memos to elaborate upon the codes and their application. Two independent coders (L.O. and M.N.) who were blinded to the data, coded the data to allow for interrater reliability. The overall percentage agreement was 98.4% and the *κ*-coefficient was 0.77, representing substantial agreement.

Thematic analysis was facilitated by immersion in the data, through multiple readings of the transcripts and memo writing to highlight emergent themes and insights. L.O., M.N. and S.M.K. reviewed the themes by closely examining the data-set and comparing themes against the data-set to come up with the final list of named and defined themes. To develop frameworks, mapping and interpretation of identified themes was then undertaken by observing and interpreting the interrelationships between themes.

## Results

Out of the 25 participants interviewed, 68% were male, and more than half of the participants were married (52%) and had post-secondary level of education (60%). The median age for the study participants was 37 years (range 22–60 years). Table 2 below provides more insight into the demographics of the participants.

**Table 2:** Sociodemographic characteristics of study participants

Characteristic	*N* = 25
Gender (male)	16
Age (years), median, mean	37, 37.9
Level of education	
No formal education	1
Primary level	6
Secondary level	3
Tertiary level	15
County of residence	
Kilifi	19
Mombasa	6
Religion	
Christian	20
Muslim	5
Prior experience with mental health services	
Provider of service	12
User of service	7
No experience	6
Marital status	
Single	8
Married	13
Separated/divorced	4

**Fig. 1:**
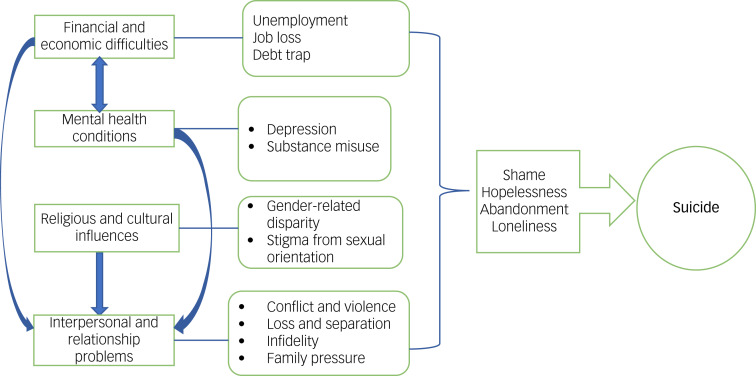
Framework for interrelatedness of reasons leading to suicidal behaviour in the Coast region.

We identified four key interrelated themes as reasons given for suicidal behaviour: interpersonal and relationship problems, financial and economic difficulties, mental health conditions and religious and cultural influences (Fig. 1). We identified six key recommendations for suicide prevention in the Coast region from our thematic analysis, ordered by participants’ emphasis: (a) increasing access to counselling services and support groups, (b) improving mental health awareness and skills training, (c) restriction of suicide means, (d) decriminalisation of suicide, (e) economic and education empowerment and (f) encouraging religion and spirituality. Details of these reasons and recommendations are outlined below. We highlight the reasons and recommendations in every section, with a quote that corresponds with our thematic analysis.

### Reasons for suicidal behaviour

#### Interpersonal and relationship problems

##### Interpersonal conflict in romantic relationships

Conflict within romantic relationships was repeatedly highlighted as a reason behind suicidal behaviour. Conflict was often related to infidelity and in some cases led to relationship breakdown, divorce and separation of the concerned individuals. Infidelity, especially if involving the female partner, would result in the male partner harbouring feelings of intense shame. For the youth, break-ups would result in feelings of betrayal, loneliness and a sense of abandonment. Emotional distress resulting from violence and abuse was also mentioned as a reason for suicidal behaviour.

‘Even for the male youth if someone realises that a lady has been unfaithful or unserious with their relationship and they had done a lot of favours for them, then they feel they are not worth living’ (female, age 30s, nurse).

‘…these middle-aged persons, the married, betrayals, extramarital affairs, the person has invested a lot may be and then they find themselves that there is another lady inside[meaning in the relationship] and they try to kill themselves because of that’ (male, age 30s, clinical officer psychiatrist).

##### Unmet personal and societal expectations

A reason specifically affecting the youth was parents and caregivers’ high and unrealistic school performance expectations. The youth would end up frustrated from these expectations and if the performance failed to measure up, the youth's distress would make them consider suicide as an escape from the pressured and stressful environment.

‘…So, one feels like the best thing is to get out of the world because nobody loves them because of undue pressure and unrealistic expectations from parents and even our education systems have contributed big time to those pressures on young people. If they get an A, then they are a special person but if they have a D, they are nothing’ (male, age 40s, pastor).

On the other hand, parents (especially the elderly) were perceived to consider suicide because of neglect and abandonment by their children. Young adults would normally leave their rural home and migrate to the cities, reducing home visits and communication. The elderly would thus feel lonely, abandoned and with a reduced sense of worth and purpose.

‘The older ones, they tend to take their lives because their children are away from them, they have abandoned them … they give the history that I have given my land I have sold my land to educate my children but now they are not there for me so why should I live I think I should die. So it`s like betrayal but now in the sense that may be they feel they have been neglected, no food for them, no care, no medication’ (male, age 30s, clinical officer psychiatrist).

##### Loss of loved one

The feeling of grief from the death of a loved one was mentioned as a contributing reason to consider suicide. This risk was perceived even higher if the loved one died as a result of suicide.

‘People who lose people who were really close to them… it depends on the situation, how the experience was… So for some it`s tragic, yah, because like what happened in my case… He committed suicide… There was no closure because you are thinking like okay this is supposed to be the best friend’ (male, age 20s, suicide attempter).

#### Financial and economic difficulties

##### Loss of income

Termination of a source of income from employment or a business (e.g. following a business collapse) was provided as a reason contributing to suicidal behaviour. Feelings of shame and guilt following loss of income especially affected men as they are perceived to be the primary source of income. Retirement was perceived to contribute to suicidality because of the lack of steady income and bringing about a sense of worthlessness, particularly in professionals who are often highly defined by their work identity.

‘Let me tell you I did a job but when I asked for payment, my employers were not happy, and they laid me off … I had worked there for seventeen years, was hardworking doing my job … So when I was alone thinking of all the things I had done, I got suicidal thoughts’ (male, age 30s, suicide attempter).

##### Unemployment

Lack of jobs and therefore a source of income was commonly mentioned as reason for some contemplating suicidality. Persons with a higher level of education who were unable to secure jobs would end up frustrated, as they were forced to be dependent on others. They felt guilty for not being supportive at home, where scarce resources were used to educate them. Closely related to the state of unemployment and affecting the older age group is the issue of retirement.

‘There are problems like lack of a job, you don't have a job and you have been looking for a job without success and when you go home the wife and children want to eat but there is no job. The wife runs away, and the outcome is that you end up committing suicide’ (male, age 30s, suicide attempter).

##### Debt trap

Reported in the interviews was that some individuals who were incapable of paying debts owed would feel trapped in accumulating debt and would contemplate suicide as a way of escaping the distress from the debt trap.

‘…he was actually simply going through financial problems, he had so many debts to the extent that he did not know how to come out of that situation. He did not want to be embarrassed or to be taken to jail so the easy thing is to end your life’ (male, age 40s, pastor).

#### Mental health conditions

##### Depression and other mental illnesses

Depression was mentioned often as a common last pathway following the other life events, such as job loss, bereavement and substance use. Persons with mental health conditions presenting with psychotic features were perceived to undergo a lot of stigma and discrimination, which in itself contributed to the individual's motivation to attempt suicide.

‘After stress comes depression, after depression comes suicide’ (female, age 20s, bereaved family member).

‘Yah, and not just depression because there are people who are suffering from different forms of mental health issues, schizophrenia, bipolar people … their loved ones don`t even seem to care much about it so it`s tough… that loneliness that comes with it… the discrimination or stigma of having a mental illness compounds the problem and you are not able to open up?’ (male, age 20s, suicide attempter).

##### Substance use problems

Participants highlighted that substance use was a common problem in the area, with heroin use being of special concern because of its highly addictive nature. Illicit drug use especially affected the youth. Drug-dependent persons would resort to stealing and selling items from their homes, leading to family discord and eventually homelessness. The desperation to fuel the drug habit and make ends meet would often increase the risk of such individuals’ involvement with sex work, thereby increasing the risk of contracting HIV and other infectious illnesses. This vicious cycle of addiction would leave the individual highly distressed, feeling abandoned, guilty, worthless and hopeless, and likely to eventually contemplate suicide.

‘But there are those you will find using the hard narcotics like heroin. When they indulge in using things drugs and they have no job, they start stealing from the family. They sell anything so that they can buy the drugs. So there comes a time when the family gives up on them and chases them away. They go to the streets where they are not used to, they are not wanted at home and even their peers … So that pressure from all ends makes them attempt suicide’ (male, age 20s, clinical officer).

‘..The other one is HIV-related cases issue because some of them actually may not be able to access the kind of medication that is required … So some of them lose hope because of even the stigma that is associated with HIV/AIDS cases and they feel that that is the end of life’ (male, age 50s, social worker).

‘The major reason is drugs, in the coast. Those people who take drugs to the extent there is a mental breakdown, or they have those withdrawal symptoms’ (male, age 40s, magistrate).

#### Religious and cultural influences

##### Gender disparities in gender-based violence and access to education

Gender disparity in education, with more focus being on the boy child, leads to high number of incidents of early marriage and pregnancy for female children. The trauma of having one's education curtailed and being forcefully married off to an older individual results in high distress and eventual suicidality in these girls. Culturally, dowry is paid off to the girl's family, hence providing an incentive, especially for families that are financially struggling. Also mentioned as affecting young girls is rape within the family unit. Because of the cultural taboos that follow incest, many families and the victim would be hesitant in reporting such cases. The mental and physical anguish of this again leads to suicidality. Finally, unwanted pregnancy in single, young girls occurring out of wedlock often results in discrimination, and in some instances, the pregnant girl being abandoned by the family because of shame. To escape the stigma and discrimination and having to deal with the financial repercussions, such individuals may even contemplate suicide.

‘ … there is still what we call “kasumba” [an outdated and biased ideology on gender roles] meaning that the girl child is not supposed to be schooled. So those families will not cater for the education of their girl child and will give her out to be married so that they get dowry. So, if the girl does not want this or is being married to a person, she doesn`t want, they can think of committing suicide’ (male, age 20s, clinical officer).

##### Discrimination regarding sexual orientation

Religious and cultural beliefs in the area prohibit and condemn same-gender relationships. Persons in same-gender relationships often live a life of secrecy, and many are cast out from their families. The shame and loneliness from this abandonment was reported to contribute to suicidal behaviour.

‘Like for us Muslims, the issue of same-sex relationships is a very bad issue. It is better to commit fornication as it is not viewed as bad as sodomisation and if this happens to a person, it can make them lose hope and even be isolated by other community members. Such persons will have been mentally killed and even his peers will isolate them’ (male, age 50s, imam).

### Opinions on recommendations for suicide prevention

Participants were asked their opinion on what could be done to reduce cases of suicide in the community based on the reasons provided. In our analysis, we identified six key thematic areas around these suicide prevention recommendations. The themes are ordered here in a way that best reflected the data as relates to participants’ emphasis and flow of the overall story. We prioritised the topics that we found most important/most effective, partially based on the experiences from the participants.

#### Increasing access to counselling services and support groups

Use of counselling services, particularly at the community level, was repeatedly recommended. Affordable counselling services were lacking in the community, forcing community members in need of mental health support to seek care at the county hospitals and from informal providers. Related to this and still at the community level, participants emphasised the benefit of encouraging support groups for bereaved family members and peer support groups for victims of suicidal attempt. Continued follow-up for persons who had attempted suicide previously was also emphasised because of the high risk of reattempt. Many recognised the role of informal providers in delivering these counselling services. Informal providers repeatedly suggested to offer mental health support in the interviews were religious leaders.

‘…The follow up should be in the community because here in the hospital they usually come when things are already worse. They have already taken the poison … ’ (male, age 30s, medical officer).

‘I think we need to have very strong counselling centres which I don't think we have in this country…so we can have even voluntary counsellors at the community level and these things will work out’ (male, age 40s, magistrate).

‘…training pastors or sheikhs or priests that they can have some information which handles counselling… to handle these cases because unfortunately most of the cases come to the church leaders whether you are an expert in that or not, they will run to you’ (male, age 40s, priest).

#### Improving mental health awareness and skills training

Open discussions on suicidal behaviour and its consequences, especially in community forums such as chief's meetings and religious meetings, was recommended. These conversations were deemed to be helpful in reducing the stigma currently associated with suicidal behaviour, and thereby help individuals not shy away from seeking help. Gate-keeper training on basic counselling skills for suicidal persons and the need for referral was also recommended. Gate-keepers mentioned were religious leaders, traditional healers and teachers. More specialised training for front-line healthcare workers on identifying and managing suicidal behaviour was additionally recommended.

‘…create that awareness which is a very good campaign… because perception of you know mental health issues being associated with witchcraft it`s not literally true. So that awareness will make people to be more receptive towards receiving help … ’ (male, age 20s, suicide attempter).

‘…They should be educated through chief's meetings or other meetings. Like the way there was campaigns on mosquito nets and malaria prevention, then suicide issues should also be included there’ (female, age 50s, nurse).

‘I think health talks like in this waiting bay or at the Maternal and Child Health clinic (MCH) because people may have come because of a different health problem but still someone among the group will benefit from the talk regarding suicide’ (female, age 50s, nurse).

‘maybe they can try to… training pastors or sheikhs or priests that they can have some information which handles counselling … ’ (male, age 40s, priest).

#### Restriction of suicide means

Some ambivalence was noted when it came to means restriction, with many not seeing the feasibility of restricting pesticides and other poisons. However, others still thought that restrictions in the sale of pesticides and prescription drugs such as sedatives may be helpful in suicide prevention. Others pointed out the need for foot patrols around the coastline and installing physical barriers on bridges to address suicidal attempts by drowning, which were perceived to be rampant in the region.

‘…it is a bit difficult because some of them are farmers, they need the pesticide but if we can regulate the supply of the pesticides. Maybe, the seller inquires a bit … to give a bit of description before you sell the item to the customer’ (male, age 30s, clinical officer psychiatrist).

‘Let's say like at the bridge there, we can make sure there are enough barriers so that it is not accessible to anyone who wants to throw themselves’ (male, age 40s, priest).

#### Decriminalisation of suicide

Stakeholders in the legislative and law enforcement space emphasised the need to have suicide decriminalised and viewed it as more of a health than legal concern. They reported that most of the time the law was not enforced in terms of an actual jail term. Instead, the suicide attempt victim, following discharge from hospital, would likely be reprimanded by the judge in court and given a warning not to reattempt suicide. Decriminalisation of suicide was deemed to also be important in destigmatising suicidal behaviour and improving access to care. However, others, including some healthcare providers and bereaved family members, felt that the current state of the law did serve as a deterrent to suicidal behaviour. They believed that the threat of a jail term would instil fear in a suicidal individual, and this fear would subsequently prevent the individual from actual attempt.

‘ … But if you tell them like if you try to take your own life, we will jail you for 2 years, don't you think you are making matters worse? If the person is depressed, you are even causing more damage to the affected person’ (male, age 30s, medical officer).

‘…To me the law is limited in so many aspects in curbing this problem because it is a mental issue’ (male, age 40s, magistrate).

#### Economic and educational empowerment

Economic empowerment through the creation of more employment opportunities, especially for the youth, was recommended. This would address financial stressors brought on by poverty and unemployment, a clearly identified risk factor for suicidal behaviour. Educational empowerment, especially targeting young girls, who are less likely to complete their education because of poverty and cultural reasons, was also recommended. Educational empowerment for girls to address the psychosocial disadvantage from early marriage and early childbearing was recommended.

‘we should also have projects that will… generate income for them and end the financial strains because many people commit suicide because of have financial constraints’ (female, age 20s, chief).

#### Encouraging religion and spirituality

Belief in a supreme power in the form of religious affiliation was deemed to be protective against suicidal behaviour. The sense of hope and meaning emphasised in religious teachings, as well as the counselling and guidance role played by religious leaders, were especially highlighted as factors in preventing suicides. Lastly, religious teachings against suicidal behaviour were additionally deemed to be a deterrent to suicide.

‘Religious centres may also help. If someone is strictly into religion, they won`t lose hope to the extent of committing suicide. The mosques and churches can support the community’ (male, age 20s, clinical officer).

## Discussion

**Fig. 2:**
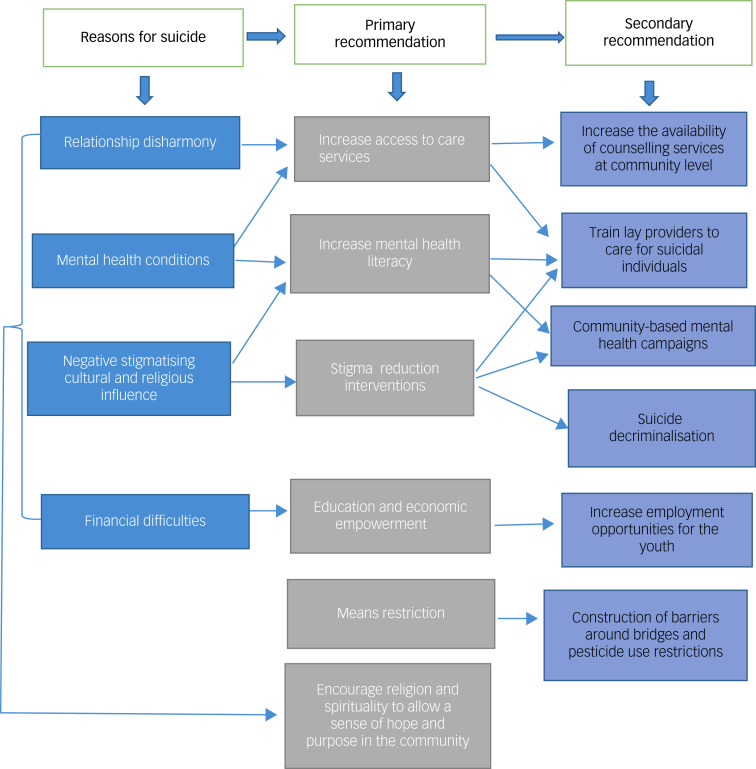
Framework on reasons for suicide and recommendations for suicide prevention. Recommendations are grouped as primary and secondary to reflect the recommendation and activities related to its implementation.

Our study findings demonstrate a multi-causal and interrelated pathway to suicidal behaviour, with influencing factors arising from interpersonal and relationship problems, financial and economic difficulties, mental health conditions and religious and cultural influences. Many of the recommendations that the participants suggested addressed these perceived reasons for suicidal behaviour (Fig. 2). For example, conflicts within relationships and mental health conditions were identified as a potential reason for suicide, whereas investment in community-based counselling services was identified as a possible solution. Notably, many suggested that these community-based services be led by trained informal providers such as clergy and other community leaders. Others emphasised the importance of peer-to-peer counselling and group counselling, especially for persons with lived experience, specifically, those bereaved by suicide or with a history of suicide attempt. Deinstitutionalisation of psychiatry services and expanding care at the community level is a key objective of the WHO, and focuses on addressing the enormous existing mental health treatment gap.^23^ In addition, non-specialist delivery of mental health services has also been highlighted as a way of addressing the low staffing ratio of mental health providers (including psychiatrists, nurses, psychologists and clinical officers) to the population served, which currently stands at 1.6 per 100 000 population in Africa compared with a global average of 13 per 100 000 population.^24^

Aside from addressing low staffing and reducing logistical barriers to care such as cost of transportation to urban facilities with psychiatry services, community-based services delivered by non-specialists addresses one key barrier to access of mental health services, namely, existing mental health and suicide-related stigma.^25^ In relation to this, our study participants revealed a hesitancy in seeking mental healthcare in established psychiatric clinics, believing that such facilities were mainly reserved for persons with serious mental illnesses and not suited for persons with moderate depression and mental distress and in need of talking therapy. Institutions that offer specialised psychiatry services are often heavily stigmatised in this study area and globally.^26^ The Kenya National Taskforce on Mental Health highlighted this stigma in their report. They documented that the stigma was mainly driven by the dilapidated and neglected state of these facilities, as well as the lack of medication and affordable counselling services.^27^ Often, because of the belief that suicidal behaviour may be linked to supernatural elements such as witchcraft or curses,^12^ some people are more likely to seek mental health-related support from traditional and faith healers.^28^ These services are arguably less effective or potentially harmful if the healer lacks a good understanding of mental health problems and when to refer severe cases.^29,30^ We found a high variability in these healers, with some having experience and expertise in suicide and others lacking training or knowledge to adequately address patients with suicidal behaviour. Stigma reduction interventions, such as increasing mental health literacy in the general community, would be an important step in destigmatising mental health and suicide, leading to increased demand for care services at the community level.^31^ Targeted training for clergy leaders and other community leaders on risk factors, assessment and management of self-harm and suicidal behaviour may be critical in equipping them with the skills needed to talk about these difficult topics.^32^ Peer and group support, as suggested by our participants, can potentially be hosted by these trained clergy and community leaders in neutral and non-stigmatising settings. Training on the need for referral for specialised care can also be imparted, to allow seriously ill individuals to access needed care. Mental health specialists do have a role in training and supervision of these paraprofessionals in addition to their work as healthcare professionals caring for referred serious psychiatric cases. For example, the Mental Health Gap Action Programme (mhGAP) training manual developed by the WHO contains a self-harm/suicide module that can be adapted for training various groups of providers.^33^ Certainly, other mental health-related factors besides the stigma of mental illness can contribute to suicidality. For instance, the symptoms experienced by persons with mental illnesses can generate distress, suffering and impairment, thus increasing suicide risk.^34^

In addition to improving mental health awareness and gate-keeper training, another strategy that addresses stigma reduction is suicide decriminalisation. Attempted suicide remains a criminal offence in Kenya, punishable by up to a 2-year jail term.^16^ Although the law is hardly enforced, criminalising an act of suicide only adds to the stigma, thereby impeding care-seeking behaviour.^35^ Our study found that the existing criminal nature of the law contributed to a reluctance to seek help and seemed to reinforce stigmatising attitudes toward survivors of suicide attempt. This corresponds with the recommendations of the WHO. The WHO emphasises that decriminalisation would not result in an increase in suicide cases, as feared by some respondents, but instead would allow more people in need of care to seek help, thereby contributing to the reduction of suicide rates.^36^ Recently, efforts toward repealing the existing law in Kenya have increased, and these efforts are being led by various stakeholders including mental health providers, persons with a history of suicidal attempt and political leaders.^37^ The involvement of various stakeholders, including politicians and legislative policy makers, is impactful and the push toward decriminalisation is promising. Even as these efforts continue, educating the public on the benefits of the decriminalisation of suicide is vital, as some still believe the law may act as a deterrent.^38^

Participants also highlighted that setting up interventions that target economic empowerment would have a considerable effect. Many participants in our study perceived financial difficulties arising from unemployment and ballooning of personal debt contributed to suicidal acts in this community. Similar to our study findings, an exploratory study in Eastern Kenya found poverty as one factor contributing to suicidal behaviour.^10^ Poverty indicators such as unemployment, debt and economic status are identified as suicide risk factors universally.^39^ Our study findings highlight three psychological constructs that could mediate the relationship between suicidal behaviour and poverty. The first one agrees with the integrated motivational volitional model,^40^ and puts forward a subjective feeling of being trapped by poverty and suicide as the only way of escaping this entrapment. The second theoretical framework supports Shneidman's model^41^ explaining suicidal behaviour as a perceived solution to an insoluble problem, which especially relates to our study findings around debt. The final framework agrees with the interpersonal psychological theory,^42^ as it brings out the subjective experience of a thwarted sense of belonging and perceived feeling of burdensomeness as a result of inability to provide for the family and meet expectations of the society. Specifically, economic empowerment strategies suggested included creation of jobs, particularly for the youth. Although not one of the identified ‘LIVE LIFE’ WHO recommendations,^4^ policies addressing economic empowerment may need greater consideration in suicide prevention interventions in sub-Saharan Africa. Economic empowerment is especially relevant in the African region, where youth aged <25 years form nearly 60% of the African population and the greater percentage of Africa's unemployed.^43^ Kenya, like many African countries, has a ‘youth bulge’, where the youth (age 18–34 years) constitute 29% of the total population.^43^ Lardier et al examined the role of intrapersonal and cognitive psychological empowerment as well as community civic engagement on drug use pattern, and found a reduction in substance use among the youth.^44^ Similarly, a positive correlation was found with economic empowerment and reduction in substance use among youth in Central Kenya.^45^ Various government economic empowerment interventions have been set up by the Kenyan Government. These offer subsidised loans, market support and linkages, and capacity building opportunities through entrepreneurship training, especially targeting the youth, women and persons with disabilities.^46^

Means restriction for suicide prevention is an important public health strategy that was suggested by most of our study participants. Specifically mentioned was placing barriers along bridges and restricting the sale of pesticide. Regional variations in commonly used suicide methods may be linked to access to certain lethal means.^47^ Local published quantitative data on suicide methods in the region supported our qualitative findings, with hanging (76%), poisoning (14%) and intentional drowning (3%) being the most commonly used methods.^6^ The coastal region is covered by several water bodies, including the Indian Ocean. Further, a large portion of the region is rural, and agriculture is one of the key economic activities. These environmental factors may increase the risk of jumping from bridges and fatal pesticide self-poisoning as suicide methods. Several pesticide restrictions have been used in various countries, from outright banning the use of highly hazardous pesticides^48^ to policies that address safe storage of pesticides.^49^ Knipe et al studied the effect of a 3-year phased ban of highly toxic pesticides in Sri Lanka and found a 21% drop in suicide rates following the ban.^50^ Structural changes to bridges to restrict access have been found to be effective in some studies.^51^ Regardless of the means restriction strategy identified, of importance is that the method identified is a high contributor to suicide in the area; the suitability of means restriction and elimination of the method should then be considered, as well as the social impact of placing such restrictions.^52^

### Interrelatedness of the reasons for suicide

In as much as we identified four core themes around suicidal behaviour, of striking importance was the interrelatedness of these themes. The perceived reasons often arose from a systemic root through to an individual level. For example, our participants perceived that poverty and unemployment, especially among the youth, could lead to financial and psychological stress. Consequently, this may then cause an individual to turn to substance use; increased frequency of substance use eventually results in drug dependence and other mental health disorders, which exacerbates the risk for suicidality. Drug dependence and added financial pressure would often then strain an individual's relationships. Later, a sense of hopelessness and entrapment into a dependence cycle, guilt from the deterioration of relationships and a loss of control from failing to provide for loved ones would then act as direct drivers of suicide and may increase the individual's mental distress, pushing them to contemplate suicide (see Fig. 1).

Other studies have similarly found a similar pattern of overlap and interrelatedness of these factors,^53^ resulting in some difficulty in teasing out specific reasons leading to a suicidal crisis. This speaks to the importance of a multipronged approach in suicide prevention strategies, and calls for more research that focuses on longitudinal elements of suicide, its causes and risk factors, and preventative strategies.

### Study strengths and limitations

This study has a number of strengths. This is the first study in Kenya to explore a community's recommendations for suicide prevention. We explored both reasons and recommendations from a diverse group of community members (including healthcare professionals and religious leaders), providing a variety of perspectives. From the study findings, we developed a framework that outlines these reasons and recommendations (Fig. 2).

Some limitations need to be considered. One limitation of this study is the failure to include focus group discussions, as information from these groups may have provided consensus in the findings. Because of COVID-19-related restrictions, we were not able to conduct focus group discussions as previously planned. However, we do believe in-depth interviews do offer a rich source of data, especially in highly stigmatised subject matters such as suicide. Second, our study only included persons residing in the Coast region of Kenya, thus our findings may not be generalisable to the rest of the country or region. Third, following a higher response rate from healthcare workers, our study did recruit a higher number of healthcare workers of varying cadre, and this unequal composition may have biased our findings or allowed for missed perspectives.

This work has been submitted as a series of two papers, with both papers analysing data from the same study cohort. Our interview guide questionnaire was structured to focus on three key areas: sociocultural perspectives of suicide, individual reasons for suicidal behaviour in the community and the community's recommendation for suicide prevention/reduction. The first paper explored how suicidal behaviour is conceptualised in this region, with a focus on sociocultural factors influencing suicidality.^12^ It showed that suicide is conceptualised as a supernatural phenomenon in this area, perceptions around suicidality differed by gender and age, and suicidal behaviour remains highly stigmatised, leading to a convoluted self-harm care pathway.

This second paper, in contrast, provides a general overview of reasons for suicidality and, unlike the first paper, captures recommendations suggested by study participants. Data emanating from this paper was analysed from the last two focus areas in our guide questions: reasons for suicidality and recommendations for suicide prevention (see Supplementary Appendix 2 for the interview guide). Some concepts in the previous paper on sociocultural factors may share similarities with this paper (e.g. aspects of stigma) and are hence addressed in both papers. Other concepts are well-outlined in the first paper and are thus not significantly expounded in this current paper, such as issues around supernatural beliefs as contributors to suicide. This was done to avoid replication/repetition of earlier submitted work.

In conclusion, this study provides an in-depth understanding behind the reasons for suicidal behaviour in the Coast region of Kenya, and explored recommendations for suicide prevention. The diverse reasons highlighted speak to the need for a multifaceted approach in addressing the suicide burden in Kenya. A greater focus on community-based interventions that include qualified training of informal providers is critical for suicide prevention. The study also highlights certain vulnerable groups that should be prioritised for such interventions, such as youth, women and persons diagnosed with mental health and substance use disorders. Future research can explore suicide-related attitudes, perceptions and care provision recommendations specifically targeting these vulnerable groups, and should design interventions that will lead to a reduction of suicide rates in Kenya (and potentially other African countries). Based on our team's experience in the clinical, research and policy mental health space, we highlight five key recommendations for suicide prevention in Kenya:

- Suicide decriminalisation: This will not only reduce the stigma related to suicidality, but will also permit suicide prevention interventions to be implemented without fear of prosecution for victims of suicide attempts. In addition, decriminalisation will likely improve access to care for these victims. Currently, many health insurance companies in the country deny cover for those who attempt suicide (based on the criminal nature of suicide), and hence this diagnosis is often not openly recorded by clinicians. This would likely affect care and accurate capture of self-harm data.
- Mental health literacy: This provides the foundation for mental health promotion, prevention and care, and can improve mental health-related outcomes such as suicide. Mental health literacy can be implemented in community spaces such as schools and religious and work institutions.
- Improve access to mental healthcare: Although some individuals may recognise that they are suicidal or dealing with mental health problems, many lack the financial resources to access specialised care. Improving access to mental healthcare means taking into consideration affordability and coverage of this care. The country is currently in the process of instituting a universal healthcare coverage that includes mental health. Also, ensuring mental health services are accessible at the community level will ensure a broader reach, so that healthcare is available for persons in need of this care. Lastly, it requires a focus on the workforce delivering these services. We recommend proper training and continued supervision to ensure a capable, qualified and culturally competent team of providers.
- Addressing social determinants of health: These often have a direct influence on an individual's mental well-being. Specifically, some determinants that need urgent addressing for suicide prevention in Kenya include unemployment and job insecurity, working life conditions and structural conflict.
- Surveillance and research on suicidality in Africa: Improving data capture of self-harm and suicide data in the country by improving case registration aids will reveal the true burden of suicidal behaviour in the country, thereby improving resource allocation. Implementation research that contextualises suicide prevention interventions is useful in prioritising and ensuring identified effective evidence-based suicide prevention interventions are scaled up and integrated into clinical practice nationally.

## Data Availability

The de-identified data that support the findings of this study are available on request from the corresponding author, L.O.

## Supplementary material

For supplementary material accompanying this paper visit http://doi.org/10.1192/bjo.2023.7. click here to view supplementary material
