# Supplementary material for: Perspectives on reasons for suicidal behaviour and recommendations for suicide prevention in Kenya: qualitative study
Source: BJPsych Open. 2023 Feb 17;9(2):e38. doi: 10.1192/bjo.2023.7 (PMC9970164; doi:10.1192/bjo.2023.7)
Supplement: Supplementary file 1 [file bjosup.zip › S2056472423000078sup002.docx]

**Appendix 4- Guide Questions for Qualitative Interviews.**

**Section 1- In-depth Interviews**

Instructions:

This form should be used for in-depth interviews

If the participants refuse to answer a question, circle the number of the question and do not mark any answers for that question. After obtaining informed consent, read the following instructions to the participants:

**“Due to different reasons, people may try to take their own lives. I would like to ask a few questions to allow us to understand how best to address this problem in the area. Please answer the questions as honestly as you can. Your information which I will write down will be kept private and this form will not have your name anywhere. All the information will be kept confidential until the conclusion of the study when it will be destroyed. If you have any questions or do not understand what I am asking you at any time, please ask for clarification.**

**Please remember that you do not have to answer any questions that you do not want to answer and you may discontinue the discussion at any time. Do you have any questions before we begin?”**

ID_______________________________________

Time_____________________________________

Date______________________________________

Name of Interviewer______________________________

County_____________________________________

Sub county _______________________________________

Village________________________________________

**Socio-Demographic Characteristics**

1. Sex Male ( ) Female ( )

2. Age in Years _____________________

3. Marital Status

Single

Currently Married

Divorced

Widow/ widower

4. Level of Education (Tick)

Never attended school

Did not complete primary school

Completed primary school but did not complete secondary school

Completed secondary school

Further studies after secondary school

Others, specify___________________________

5. Main occupation (Tick)

Peasant farmer

Small business (kiosk, kibanda)

Big business (shop)

Housewife

Salaried worker (teacher, police, chief)

Fisherman

Casual labourer

Others, specify________________________

6. Religion (Tick)

Christian

Islam

Non-practicing

Others, specify__________________________

7. Prior experience with mental health services

Provider

Family member

User of service

Caregiver

No experience at all.

**Actual Interview**

**Exploring Risk Factors**

1. **What are some of the reasons someone would try to take their own life?**

**Probe**

- 1. **For difference in risk factors by gender and age. For example: do these reasons differ if someone is male or female, old or young?**
  2. **Probe whether there is a specific group of people or specific characteristics of a person that make them more likely to take their own lives.**

**Exploring Cultural Perspective of Suicide**

1. **These people who attempt to take their own life, or who unfortunately take their own life, how are they perceived by the community?**
   1. **Probe if these perceptions differ if say one is male or female, from a certain tribe or ethnicity, from a specific religion, of a certain age (old or young).**

**Exploring Suicide Prevention**

1. **Do people who try to take their own life seek any help? If yes, where do they seek help?**
2. **Do you think that people who try to take their own lives can be helped?**

**If yes, how can they be helped? (If they give multiple options ask, what of those do you think would be most appropriate for this setting and why)**

**If they say no to the help question, then probe why they think it’s a no.**

1. **What can we do to reduce suicide in the community?**

**Probe further along these themes. For example**

- 1. **Say by addressing certain suicide methods/means/places.**
  2. **Say by addressing care**
  3. **Say be addressing religion**
  4. **Say be addressing the law.**

**THANK YOU VERY MUCH FOR YOUR COOPERATION.**

**Do you have any additional comments or questions that were not covered in the interview?**

Post Interview comment

In this part of the interview the interviewer should write notes that detail his/her feelings, interpretations and other comments. This should be done immediately after conducting the interview.
