# Supplementary material for: Perspectives on reasons for suicidal behaviour and recommendations for suicide prevention in Kenya: qualitative study
Source: BJPsych Open. 2023 Feb 17;9(2):e38. doi: 10.1192/bjo.2023.7 (PMC9970164; doi:10.1192/bjo.2023.7)
Supplement: Supplementary file 1 [file bjosup.zip › S2056472423000078sup001.doc]

**KEMRI Wellcome Trust Research Programme: Patient Information Sheet and Consent Form**

**ICF for Qualitative Study-IDIs**

***STUDY TITLE:*** Risk Factors and Socio-cultural Perspectives related to Suicidal Behavior at the Kenyan Coast.

***LAY TITLE:*** Risk Factors and Experiences related to Suicidal Behavior at the Kenyan Coast.

| KEMRI CCR | Linnet Ongeri |
| --- | --- |
| KEMRI Wellcome Trust | *Charles Newton, Symon Kariuki, Mary Bitta. (List names of investigators in a row for each institution to minimize space taken)* |
| Vrije University | *Brenda Penninx* |
| Tergooi Hospital | *Chris Schubart* |
| Amsterdam UMC/VU University | *Dr. Joeri Tijdink* |

**Who is carrying out this study and what is this study about?**

- This study is being carried out by KEMRI in collaboration with Vrije University. KEMRI is a government organization that carries out medical research to find better ways of preventing and treating illness in the future for everybody’s benefit.
- Sometimes research involves only asking questions of patients, their parents, community members or health providers about what they know, feel or do.
- In this research, we want to learn more about about suicide at the Kenyan Coast and what would make people want to end their lives by interviewing you and listening to your ideas and opinions on this. We would like to hold discussions with a total of atleast 30 participants who live at the coast of Kenya. We would like to talk to people individually.

**Why do you want to talk to me and what does it involve?**

- *Explain selection*

We feel that your experience as a person living at the Coast of Kenya and a stakeholder with an understanding of the cultural and social issues can contribute much to our understanding and knowledge of suicide in the mentally ill.

- I would like to ask you a number of questions about your opinion on reasons for suicidal behaviors, persons likely to attempt suicide or die from suicide, methods used more commonly in the area in attempts or completed suicide, cultural and religious implication of suicidal behavior as well as available care. If you do not want to answer any of the questions you may say so and the interviewer will move on to the next question. The discussion will take place in a private space at a venue that is conducive and convenient to you. No-one else but the interviewer will be present unless you would like someone else there .
- For interviews conducted remotely over the phone we shall take a short break if you have any external interruptions or are losing concentration. If the interview is interrupted, we shall reschedule the remainder of the session as soon as possible, ideally on the same day.
- The discussion will be recorded in a note book and also audio recorded to assist later in fully writing up the information. No-one will be identified by name in the recording.

**Are there any risks or disadvantages to me / my child of taking part?**

- The discussions should take approximately 1 hour. You will be provided with 350Ksh to cover for the cost of fare and time.
- There are minimal risks associated with participating in a qualitative interview. Questions asked during the interview might cause you to reflect on your emotions and/or contact(s) with mental health care, and these thoughts and feelings may be upsetting to you.
- You are free to decline to answer any questions, or to leave the meeting at any time. If you feel that you are becoming distressed, please let your interviewer know and the session will be stopped and you will be provided with assistance. If you appear to be distressed at any point, the interviewer may stop and try to find out more about your feelings, in order to refer you for psychosocial help, as necessary. This may include referral to mental health care.
- Transport cost for this referral will be met by study funds.

**Are there any advantages to me taking part?**

There are no individual benefits to taking part. In talking to us, you will contribute to the knowledge of suicide that may help other people in Kenya and elsewhere in the future, for example through developing new health policies.

**Who will have access to the information I give?**

- All of our documents/ recordings are stored securely in locked cabinets and on password protected computers. The knowledge gained from this research will be shared in summary form, without revealing individuals’ identities.
- In future, information collected or generated during this study may be used to support new research by other researchers in Kenya and other countries on mental illness and suicide related behavior. In all cases, we will only share information with other researchers in ways that do not reveal individual participants’ identities. For example, we will remove information that could identify people, such as their names and where they live, and replace this information with number codes. Any future research using information from this study must first be approved by a local or national expert committee to make sure that the interests of participants and their communities are protected.
- All audio recordings will be destroyed after transcription.

**Who has allowed this research to take place?**

All research at KEMRI has to be approved before it begins by an accredited IRB committee who look carefully at planned work. They must agree that the research is important, relevant to Kenya and follows nationally and internationally agreed research guidelines. This includes ensuring that all participants’ safety and rights are respected.

**What will happen if I refuse to participate?**

All participation in research is voluntary. You are free to decide if you want to take part or not. If you do agree you can change your mind at any time without any consequences.

**What if I have any questions?**

You are free to ask me any question about this research. If you have any further questions about the study, you are free to contact the research team using the contacts below:

Dr. _Linnet Ongeri_, KEMRI Wellcome Trust Research Programme, P.O. Box 230, Kilifi. Telephone: [Insert mobile 0722615999] or 0722 203417, 0733 522063, 041 7522063

**If you want to ask someone independent anything about this research please contact:**

Community Liaison Manager, KEMRI Wellcome Trust Research Programme, P.O. Box 230, Kilifi. Telephone: 041 7522 063, Mobile 0723 342 780 or 0705 154 386

***And***

The Head, KEMRI Scientific and Ethics Review Unit, P. O. Box 54840-00200, Nairobi; Telephone numbers: 0717 719477; 0776 399979 Email address: [seru@kemri.org](mailto:seru@kemri.org)

**KEMRI-Wellcome Trust Research Programme consent form for [Study Title]**

I have had the study explained to me. I have understood all that has been read/explained and had my questions answered satisfactorily. And I agree to take part in this research.

**I agree for the interview/discussion to be recorded  Yes  No**

I understand that I can change my mind at any stage and it will not affect me in any way.

| **Signature:** |  | **Date:** |  | |
| --- | --- | --- | --- | --- |
|  |  | | | |
| **Participant/guardian Name:** |  | **Time:** | |  |
|  | ***(please print name)*** |  | | |

--------------------------------------------------------------------------------------------------------------------------------------

***Where participant agreed to remote interview and verbal consent:***

I attest that the information concerning this research was accurately explained to and apparently understood by the study participant and that informed consent was freely given by the study participant.

**Witness’ signature:** _____________________________________ **Date** _____________

**Witness’ name**: _____________________________________ **Time** ______________

**A witness is a person who is independent from the study or a member of staff who was not involved in gaining the consent.*

Thumbprint of the parent as named above if they cannot write:

------------------------------------------------------------------------------------------------------------------------------------------

***[Following section is recommended, and where verbal consent is obtained, must be signed by person undertaking informed consent.]***

I have followed the study SOP to obtain consent from the [participant/guardian]. S/he apparently understood the nature and the purpose of the study and consents to the participation [of the child] in the study. S/he has been given opportunity to ask questions which have been answered satisfactorily.

**Designee/investigator’s signature:** ____________________________ **Date** ____________

**Designee/investigator’s name :** _____________________________**Time** ____________

(Please print name)

# THE PARENT/GUARDIAN SHOULD NOW BE GIVEN A SIGNED COPY TO KEEP

*……………………………………………………………………………………………………………………………………………………………………………*

***SHIRIKA LA UTAFITI LA KEMRI WELLCOME TRUST: FOMU YA MAELEZO NA IDHINI***

**Kichwa Rahisi Cha Utafiti**: Sababu zinazochangia watu kutaka kujitoa uhai pamoja na maoni ya watu kuhusu jambo hili katika pwani la Kenya.

| Taasisi | Watafiti |
| --- | --- |
| KEMRI CCR | *Linnet Ongeri (PI)* |
| KEMRI-Wellcome Trust | *Charles Newton(Co-Pi), Symon Kariuki, Mary Bitta* |
| Vrije University | Brenda Penninx |
| Tergooi Hospital | Chris Schubart |
| Amsterdam UMC/VU University | Joeri Tidjink |

**Ni nani anayefanya utafiti huu?**

- Utafiti huu unafanywa na KEMRI. KEMRI ni shirika la serikali linalofanya utafiti wa afya ili kutafuta njia bora za kukinga na kutibu magonjwa kwa siku za usoni kwa manufaa ya watu wote.
- Wakati mwengine utafiti unahusisha kuuliza maswali pekee kwa wagonjwa, wazazi wao, jamii au wahudumu wa afya kuhusu wanachojua, wanachohisi na wanachofanya.

**Utafiti huu unahusu nini?**

- Katika utafiti huu, tunataka kujifunza zaidi kuhusu matatizo yanayoambatana na mtu kutaka kujitoa uhai. Na ni mambo gani yanayowezachangia mtu kuwa na mawazo haya au kitendo hiki. Tungependa kuelewa maoni ya watu wanaoishi hapa Pwani kwa kukuhoji kutumia maswali machache.
- Tutashirikisha takriban watu 30.
- Tunakuhusisha katika utafiti huu kwa sababu wewe ni mkaaji hapa pwani Kenya na mshika dau kwa mambo ya afya.

**Itahusisha nini** kwa wale watakaojiunga na utafiti??

Tunaamini ya kwamba maoni yako kama mtu anayeishi hapa Pwani na ameugua magonjwa ya kiakili ni muhimu na yanaweza kutuwezesha kuelewa kwa undani chanzo cha shida hizi.

- Ningependa kukuuliza maswali kadhaa kuhusu maoni yako kuhusu sababu mtu angeamua kujitoa uhai, njia ambazo hutumika sana sana katika eneo hii, unyanyapaa kuhusu swala hili. Pamoja na huduma bora ya afya ambayo yaweza kuwasaidia watu hawa.
- Kuna uwezekano wa wewe kuhisi usumbufu wakati unapojibu maswali kukuhusu. Hauna lazima ya kujibu kila swali ikiwa haujisikii huru. Uko na uhuru wa kuchukua kipindi kidogo cha mapumziko.
- Mahojiano haya yataendelezwa katika chumba faraghani hapa hospitalini. Hakuna yeyote mwingine atakuwepo isipokuwa anayekuhoji. Lakini ukipenda mwenzako kuwepo ana huru ya kujiunga nawe.
- Kwa mahojiano yatakayofanywa kwenye mtandao wa simu. Una uhuru wa kupumzika na kuendelea na mahojiano wakati unaokufaa.
- Mahojiano yatarekodiwa katika kitabu na kinasa sauti ili kuwezesha maoni yote kutambulika katika kumbukumbu. Hakuna atakaye tambulika kwa jina katika rekodi hiyo.

**Je kuna madhara au usumbufu wowote kwa wale watakaoshiriki?**

- Mahojiano yatachukua muda wa takriban saa moja. Utapewa nauli ya shilling 350.
- Kuna madhara chache yanayoweza kutokana na kushiriki katika mahojiano. Maswali yatakayo ulizwa yaweza kukufanya utafakari kuhusu hisia au mawasiliano na huduma ya afya ya akili. Mawazo na hisia hizi yaweza kukufadhaisha.
- Una haki ya kutojibu maswali yoyote, au kuacha mkutano wakati wowote. Ukihisi kuhangaika, tafadhali mueleze anayekuhoji na kikao kitasimamishwa na utasaidiwa. Ikiwa utaonekana kufadhaika, anayekuhoji atasimamisha kikao na kujaribu kujua Zaidi juu ya hisia zako ili akuelekeze vizuri ambapo unaweza ukapata huduma. Hii yamaanisha yaweza ukaelekezwa utakapopata huduma ya kiakili.
- Utafiti huu utasimamia gharama ya usafirishaji ya rufaa hii.

**Je kuna manufaa yoyote kwa wale watakaoshiriki?**

•	Hakuna manufaa ya kibinafsi kwa kushiriki.

•	Hata hivyo, matokeo ya utafiti huu yatasaidia wanasayansi kujifunza zaidi mambo kuhusu hisia za kutakakujitoa uhai na kuelewa zaidi matatizo ya kiakili.

•	Tunatarajia ya kuwa haya yote yataboresha matibabu siku za usoni.

**Ni nani atakayeweza kufikia habari ninazopeana?**

- Stakabadhi/rekodi zetu zote zinahifadhiwa salama kwenye kabati zilizofungwa na komputa zinazohitaji neno maalum kufunguliwa. Ufahamu utokanao na utafiti huu utasambazwa kwa ufupi, bila ya kufunua vitambulisho vya kibinafsi.
- Ijapokuwa hatutawapa watafiti jina lako, tutawapa habari za jumla kukuhusu kama vile kabila, sehemu unayotoka, miaka na jinsia. Habari hizi huenda zikawasaidia watafiti kuchunguza ikiwa vyanzo vya matatizo fulani ya kiafyia zinafanana katika makundi tofauti ya watu. Kuna uwezekano wa matokeo hayo kuja kusaidia watu katika kundi moja na wewe.
- Baadaye, utafiti wowote utakaohitaji rekodi hizi, utalazimika kupata ruhusa kutoka kwa kamati ya utafiti ili kuhakikisha utafiti huo umetekelezwa kwa njia ya sawa.
- Rekodi ya kinasa sauti itafutwa baada ya kuwekwa kwenye kumbukumbu.

**Ni nani ameidhinisha utafiti huu ?**

Tafiti zote za KEMRI ni lazima ziidhinishwe kabla zianze na kamati zenye kibali ya utafiti zinazoangalia kwa makini mpango wa kazi. Ni lazima wakubali kwamba utafiti ni muhimu na wafaa Kenya na unafuata muongozo unaokubalika kitaifa na kimataifa. Hii ni pamoja na kuhakikisha kwamba usalama na haki za washiriki wote zimeheshimiwa.

Nini kitafanyika nikikataa kushiriki?

•	Kushiriki kote katika utafiti ni hiari. Uko huru kuamua ikiwa ungependa kushiriki au la. Ukikubali unaweza kubadilisha nia wakati wowote bila ya madhara yoyote.

•	Unaposhiriki katika utafiti huu, una uhuru wa kubadilisha nia wakati wowote na kuamua kutoshiriki tena.

Je nikiwa na maswali yoyote ?

Uko huru kuniuliza swali lolote kuhusu utafiti huu. Ukiwa na maswali yoyote zaidi kuhusu utafiti huu, uko huru kuwasiliana na kundi la utafiti ukitumia anwani ifuatayo.

Dkt. Linnet Ongeri, Shirika la utafiti la KEMRI Wellcome Trust, S. L. Posta 230, Kilifi. Simu: [0722615999] au Khamisi Katana, Simu: 07112677 50

**Ukitaka kumuuliza mtu huru kuhusu utafiti huu tafadhali wasiliana na**

Meneja wa kitengo cha uhusiano mwema na jamii, KEMRI Wellcome Trust Research Programme, S. L. P 230, Kilifi. Simu: 0723 342 780 au 041 7522 063

Na

Katibu, Kitengo cha kukagua sayansi na maadili cha KEMRI, S. L. P. 54840-00200, Nairobi, nambari ya simu : 020 272 2541 Rununu : 0722 205 901 au 0733 400 003

Fomu ya idhini ya shirika la utafiti la KEMRI Wellcome Trust kwa utafiti wa “ Sababu zinazochangia watu kutaka kujitoa uhai pamoja na maoni ya watu kuhusu jambo hili katika pwani la Kenya.”

Nimeelezwa utafiti huu. Nimeelewa yote niliyosomewa/elezwa na maswali yangu yamejibiwa kikamilifu.

Nakubali kushiriki kwenye utafiti huu.

Ninaelewa kwamba naweza kubadilisha nia wakati wowote na haitaniathiri kwa njia yoyote

Sahihi : ______________________________________________Tarehe : __________________

Jina la mshiriki/mlezi : ___________________________________Saa : ____________________

(Tafadhali andika jina kwa herufi kubwa)

--------------------------------------------------------------------------------------------------------------------------------------

**Iwapo mshiriki amekubali kuhudhuria utafiti kwa kutumia mtandao wa simu na amepatiana idhini ya maneno, hakikisha shahidi* anafuatilia utaratibu wa utafiti na aweke sahihi hapo chini:**

Ninathibitisha kwamba habari kuhusu utafiti huu zimeelezwa kwa usahihi na bila shaka zimeeleweka na mshiriki na kwamba idhini itokanayo na kuelewa ujumbe ilipeanwa bila kushurutishwa.

**Sahihi ya Shahidi________________________________________Tarehe:__________________**

**Jina la Shahidi____________________________________________Saa:________________**

*Shahidi ni mtu ambaye yuko huru kutokana na utafiti au mfanyajikazi ambaye hakuhusika katika kupata idhini

Alama ya kidole gumba cha mshiriki kama alivyotajwa hapo juu ikiwa hawezi kuandika

**Sehemu ifuatayo imependekezwa, na ikiwa idhini bila ya maandishi itaombwa, sharti iwekwe sahihi na mtu anayeomba idhini**

Nimefuata taratibu zote za kuomba idhini katika utafiti huu kutoka kwa mshiriki. Ni wazi kwamba ameelewa hali na lengo la utafiti, na amepeana idhini ya kushiriki katika utafiti. Mshiriki amepewa nafasi ya kuuliza maswali ambayo yamejibiwa kikamilifu.

**Sahihi ya Mtafiti mwakilishi/Mtafiti:______________________________Tarehe:__________________**

**Jina la Mtafiti Mwakilishi/Mtafiti:________________________________Saa:_____________________**

[Tafadhali andika jina kwa herufi kubwa]

**MSHIRIKI/MZAZI/MLEZI SASA APEWE NAKALA ILIYOWEKWA SAHIHI AHIFADHI**
